# Matching Graft Quality to Recipient’s Disease Severity Based on the Survival Benefit in Liver Transplantation

**DOI:** 10.1038/s41598-020-60973-9

**Published:** 2020-03-05

**Authors:** Audrey Winter, Cyrille Féray, Corinne Antoine, Daniel Azoulay, Jean-Pierre Daurès, Paul Landais

**Affiliations:** 1 University of Montpellier, Department of Biostatistics, UPRES EA2415, Clinical Reasearch University Institute, Montpellier, France; 2 Beau Soleil Clinic, Languedoc Mutualité, Montpellier, France; 3 Department of Radiological Sciences, Medical Imaging & Informatics, University of California, Los Angeles, CA USA; 4 Centre Hépato-Biliaire, INSERM 1193, Paul Brousse Hospital, Villejuif, France; 5 Agence de Biomédecine, Saint-Denis, France

**Keywords:** Statistics, Epidemiology, Liver cancer, Liver cirrhosis

## Abstract

Persistent shortage and heterogeneous quality of liver grafts encourages the optimization of donor-recipient matching in liver transplantation (LT). We explored whether or not there was a survival benefit (SB) of LT according to the quality of grafts assessed by the Donor Quality Index (DQI) and recipients’ disease severity, using the Model for End-Stage Liver Disease (MELD) in 8387 French patients wait-listed between 2009 and 2014. SB associated with LT was estimated using the sequential stratification method in different categories of MELD and DQI. For each transplantation, a stratum was created that matched one transplanted patient with all eligible control candidates. Strata were thereafter combined, and a stratified Cox model, adjusted for covariates, was fitted in order to estimate hazard ratios that qualified the SB according to each MELD and DQI sub-group. A significant SB was observed for all MELD and DQI sub-groups, with the exception of high MELD patients transplanted with “high-risk” grafts. More specifically, in decompensated-cirrhosis patients, “high-risk” grafts did not appear to be detrimental in medium MELD patients. Interestingly, in hepatocellular-carcinoma (HCC) patients, a significant SB was found for all MELD-DQI combinations. For MELD exceptions no SB was found. In terms of SB, “low-risk” grafts appeared appropriate for most severe patients (MELD > 30). Conversely, low/medium MELD and HCC patients presented an SB while allocated “high-risk” grafts. Thus, SB based matching rules for LT candidates might improve the survival of the LT population as a whole.

## Introduction

Liver transplantation (LT) is the only lifesaving therapy to cure end-stage liver disease and hepatocellular carcinoma (HCC). Organ shortage is one of its most limiting factors^1^. Persistent shortage of donors has led to “low-quality” or “high-risk” grafts, which are often associated with a higher risk of graft loss^2^.

One of the main impediments to the use of these grafts is that guidelines for allocations are not clearly established and can vary between transplant centers and countries^3–5^. If misused, these grafts could have a negative impact on patient survival and generate a significant increase in re-transplantation. However, in contrast, they could reduce the risk of death on the waiting list (WL) and offer a favorable risk-benefit ratio^6^.

To assess the quality of liver grafts, scores have been developed such as the Donor Risk Index (DRI) by Feng *et al*.^7^ using the Organ Procurement and Transplantation Network database. In previous work^8^, we performed an external validation of this score on our database, as well as the Eurotransplant-Donor Risk Index (ET-DRI)^9^, according to the methodology proposed by Royston and Altman^10^. We showed that neither the DRI nor the ET-DRI could be validated^8^. In order to qualify the grafts, we then developed a new donor risk score, the Donor Quality Index (DQI), for which both internal and external validation have been performed^11^.

In France, the “Agence de la Biomédecine” (ABM) is responsible for managing the WL and allocating the grafts (see Supplementary Information for more details). The French allocation system, as in most countries, is based on a “sickest first” policy, according to the “Score foie”^12^. This score takes into account the Model for End-Stage Liver Disease (MELD)^13^, and other criteria, such as the waiting time, and distance between donor and recipient. In this allocation system, the only existing matching criterion between donors and recipients is the ABO group.

Due to the lack of guidelines to optimize the matching between donor and recipient, this process is not standardized and depends on a number of factors, including transplant center policies, characteristics of the local WL including waiting times, and physician expertise. Because the sickest (i.e. with high MELD) candidates are the most likely to have difficult post-operative periods, high-risk (low-quality) grafts should not be considered for this population. But in the absence of any matching rules, “low-risk” patients can often be matched with “low-risk” or “high-quality” grafts, leaving fewer grafts available for the sickest patients.

The rate of discard and non-recovery rates remains high and the appropriate use of grafts could therefore reduce the consequences of organ shortage. What kind of matching would be optimal? Would it be beneficial to remain on the WL and possibly later receive a “better-quality” or “lower-risk” graft? However, this practice would expose a candidate on the WL to clinical deterioration or even death. Using the French LT database, we explored the optimal matching between grafts’ quality defined by the DQI, and recipients’ disease severity status determined by the MELD, in the whole LT population studied, along with specific indications, namely, decompensated-cirrhosis, HCC and MELD exception. The matching was assessed by the survival benefit (SB) defined according to a sequential stratification modeling^14–17^.

## Material

### Data

Our study included all adult LT candidates on the French waiting-list between 2009 and 2014 who were followed prospectively under the control of the ABM, together with all donors after brain-death registered over the same period. Information related to wait-listed patients in France between January 4, 2009 and December 31, 2014 was obtained from the ABM (https://www.agence-biomedecine.fr/Organes). Of note, in France, during the 2009–2014 period, the proportion of discarded grafts (i.e. collected but not transplanted) ranged from 5 to 7% (Table 1S in Supplementary Information, provides the proportion per year and reasons why those grafts were not transplanted). The study was conducted with the approval of an Independent Ethics Committee (Articles L 1121-1 to L 1126-11 of the Code of Public Health). Authorization was also obtained from the “Commission Nationale de l’Informatique et des Libertés” (agreement No. 915206). Data were obtained from ABM without identifiers.

**Figure 1:**
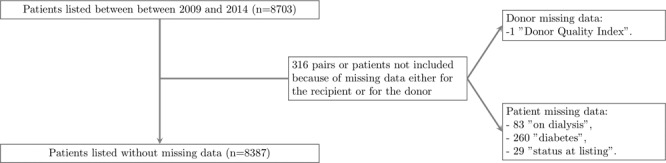
Flow chart detailing missing data for patients wait-listed and/or transplanted between January 2009 and December 2014 and the corresponding donors.

LT candidates under 18 years of age or with multiple organ transplants were not included. Candidates with incomplete data were not retained as specified in the flow diagram presented in Fig. 1. In total, 8387 patients waiting for a deceased donor LT were analyzed (Table 1).

**Table 1:** Candidate characteristics at listing, recipients and donor characteristics at liver transplantation (LT).

Recipients characteristics	At listing (N = 8387)	At LT (N = 5753)
Age	53.6 (10.4), 55.5	53.8 (10.6), 55.6
Female	2175 (26.0%)	1505 (26.2%)
Body mass index	25.8 (4.9), 25.2	—
Decompensated cirrhosis	3246 (38.7%)	2319 (40.3%)
Hepatocellular Carcinoma	2951 (35.2%)	1965 (34.2%)
Hepatitis C	1940 (23.1%)	1239 (21.5%)
Hepatitis B	481 (5.7%)	353 (6.1%)
Alcoholic cirrhosis	4225 (50.4%)	2810 (48.8%)
Medical urgency	574 (6.8%)	455 (7.9%)
Diabetes	1925 (23.0%)	1318 (23.0%)
Previous transplantation	805 (9.6%)	586 (10.2%)
On dialysis	442 (5.3%)	—
Status:		
Intensive Care Unit	1391 (16.6%)	1157 (20.1%)
Hospital	1105 (13.2%)	780 (13.6%)
Home	5891 (70.2%)	3816 (66.3%)
ABO group:		
A	3598 (42.9%)	2575 (44.8%)
AB	330 (3.9%)	250 (4.3%)
B	949 (11.3%)	653 (11.4%)
O	3510 (41.9%)	2275 (39.5%)
Model for End-stage Liver Disease (MELD)	19.0 (10.0), 17.0	20.9 (11.0), 19.0
MELD group at risk:		
Low MELD, 6 ≤ MELD ≤ 15	3813 (45.5%)	2294 (39.9%)
Medium MELD, 15 < MELD ≤ 30	3188 (38%)	2087 (36.3%)
High MELD, MELD > 30	1386 (16.5%)	1372 (23.8%)
MELD exception	1178 (14.0%)	1016 (17.7%)
Waiting time (in days)	228.7 (312.7), 123	175.7 (213.7), 104
Death	1066 (12.7%)	1329 (23.1%)
Graft loss	—	398 (4.7%)
Removed from the waiting list due to worsening of patient’s condition	717 (8.5%)	—
Removed from the waiting list due to improving of patient’s condition	511 (6.1%)	—
**Donors characteristics (N = 5753)**		
Donor age > 69 years		1438 (25%)
Cause of death:		
Anoxia		708 (12.3%)
Trauma		1403 (24.4%)
CVA		3483 (60.5%)
Other		159 (2.8%)
Intensive care unit stay > 4 days		1171 (20.4%)
ABO group:		
A		2513 (43.7%)
AB		203 (3.5%)
B		559 (9.7%)
O		2478 (43.1%)
MDRD creatinine clearance: lowest (ml/min/1.73 m^2^):		
≥90		1613 (28.0%)
<60		2034 (35.4%)
60–90		2106 (36.6%)
Liver split		267 (4.6%)
Donor Quality Index (DQI)		1.85 (0.5)
DQI group at risk:		
Low DQI, 1.0 < DQI ≤ 1.58		1638 (28.5%)
Medium DQI, 1.58 < DQI ≤ 2.35		3149 (54.7%)
High DQI, 2.35 < DQI		966 (16.2%)
Cold ischemia time (in minutes)		495.2 (267.3), 468

Mean, standard deviation and median are reported for quantitative covariates; number and percentage are reported for qualitative covariates.

Decompensated-cirrhosis was identified using the MELD score and the Child-Pugh score. All patients presenting with cirrhosis, without HCC or non-HCC liver cancer, a MELD score ≥ 16 and a B or C Child-Pugh score were considered as decompensated-cirrhosis. Diagnosis of HCC was performed according to Amin *et al*.^18^. MELD exceptions were identified and resulted in extra points while on the WL^19^. In France, MELD-exceptions (i.e. extra points) are typically given when patients meet one of two conditions: (1) they have a diagnosis of one of roughly 20 specified rare diseases (amyloidosis, polycystic liver, etc.); or (2) they are experiencing severe complications of cirrhosis (refractory ascites, chronic encephalopathy, etc.).

## Results

**Figure 2:**
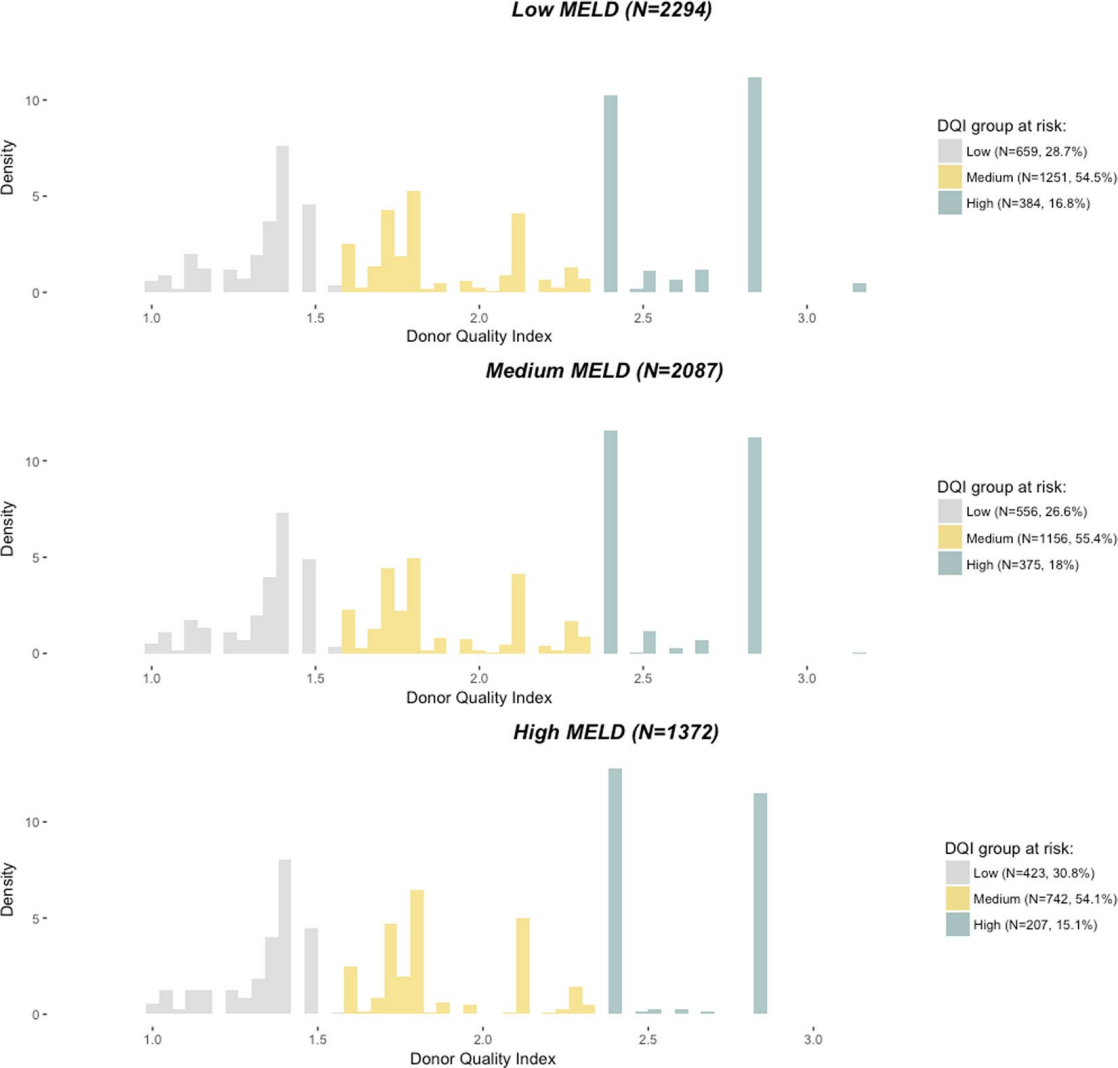
Distribution of the 5753 liver transplantations by categories of Model for End-stage Liver Disease (MELD) at transplantation and Donor Quality Index (DQI) (${\chi }^{2}$ test, *p* = 0.048). With low DQI: 1.0 < DQI ≤ 1.58, medium DQI: 1.58 < DQI ≤ 2.35, high DQI: 2.35 < DQI, low MELD: 6 ≤ MELD ≤ 15, medium MELD: 15 < MELD ≤ 30 and high MELD: 30 < MELD.

Among the 8387 candidates, 5753 had received liver transplants. Candidate, recipient and donor characteristics are shown in Table 1. The distribution of LT by MELD and DQI categories appears on Fig. 2.

### Sequential stratification: survival benefit by MELD and DQI categories

**Figure 3:**
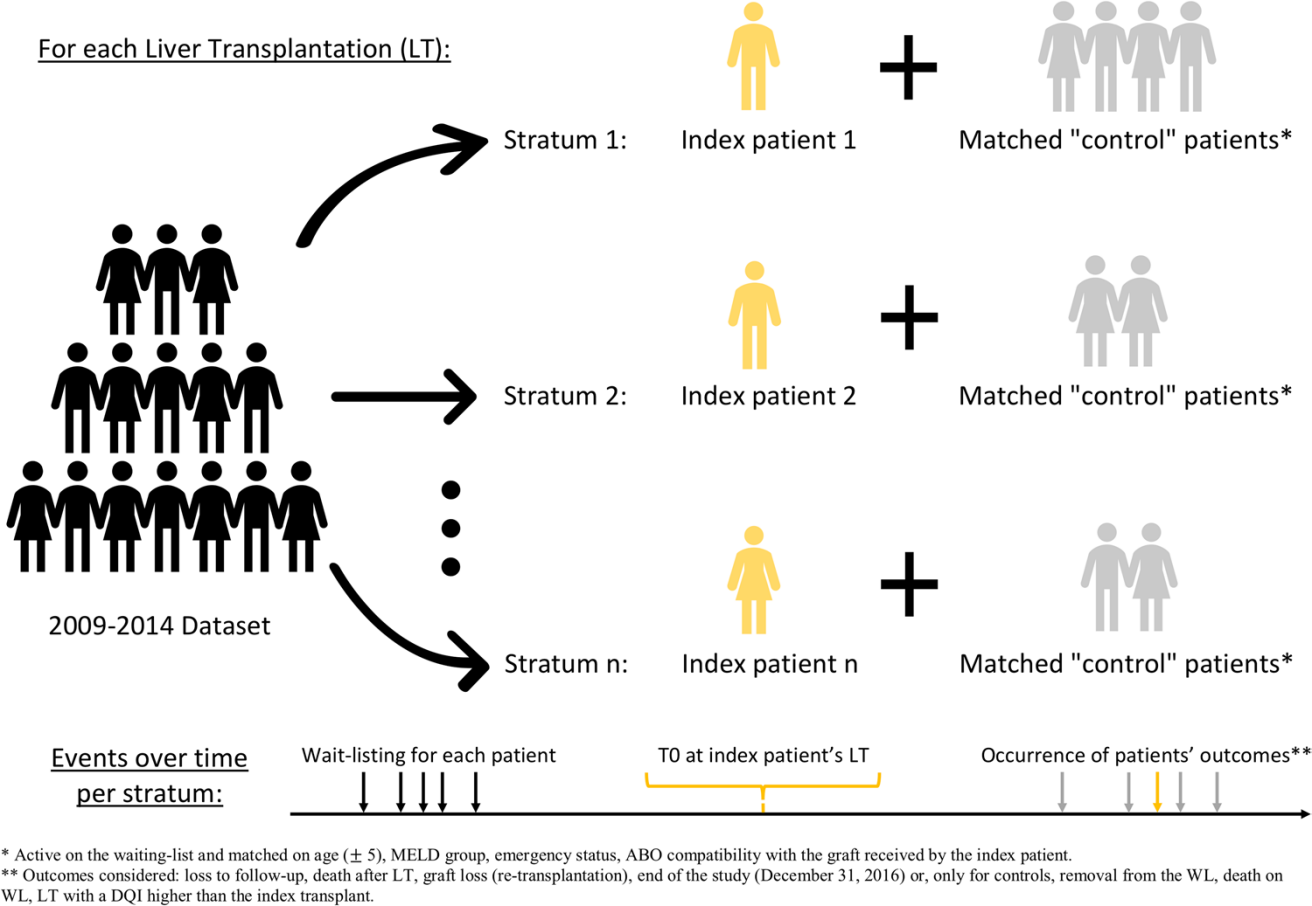
Reorganization of the observed data in order to be as close as possible to randomized controlled trial data.

A stratum was created that matched one transplanted patient (called the “index patient”, Fig. 3) with all eligible control candidates (according to the criteria detailed in the Methods section), for each of the 5753 LT in the dataset. Control patients were chosen among all candidates, excluding the index patient of the retained stratum (N = 8386). A patient can therefore be the index in one stratum and a control in another. If no control patient was matched to an index patient, the stratum was dropped, and the index patient was not included in the analysis as an index patient (though this patient may have been included as a control patient in another stratum). In all, 4754 strata were created, which were combined and fit with a stratified Cox regression model. The SB was estimated through hazard ratios (HRs) in each MELD and DQI category.

**Figure 4:**
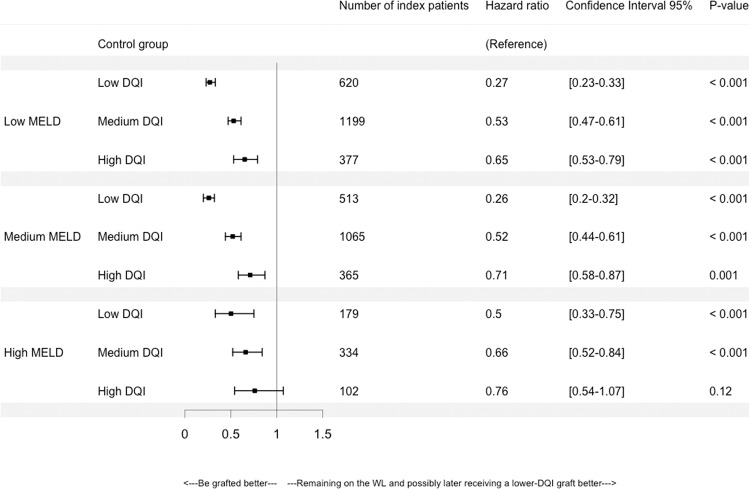
Hazard ratios according to Model for End-stage Liver Disease (MELD) at transplantation and Donor Quality Index (DQI) categories. With low DQI: 1.0 < DQI ≤ 1.58, medium DQI: 1.58 < DQI ≤ 2.35, high DQI: 2.35 < DQI, low MELD: 6 ≤ MELD ≤ 15, medium MELD: 15 < MELD ≤ 30 and high MELD: 30 < MELD. The reference is the control group that consists of patients who remained on the waiting-list (WL) waiting for a potential graft of “lower risk” or “better quality” (i.e. a ≤DQI graft) than the one of the index patient.

There was a significant SB for transplanted patients compared to patients who remained on the WL, waiting for a potential graft of “better-quality” (i.e. ≤DQI graft) for all categories, with the exception of high MELD patients who received a high DQI (“low-quality”) graft. In Fig. 4, we note that the SB is similar for patients with low or medium MELD.

The normality assumption was consistent whereas the consistency over time assumption was not. More details about the verification of the model assumptions are available in Supplementary Results and Tables 2S and 3S in Supplementary Information.

### Survival benefit in decompensated-cirrhosis, HCC and MELD exception patients

We studied MELD-DQI matching in three main indications: decompensated-cirrhosis, HCC and MELD exception, using the method described above with a supplementary matching based on LT indication (see Methods section for more details). Of the 4381 low and medium MELD recipients (Fig. 2), 1793 were HCC (without MELD exception component) and 1002 were MELD exceptions. From these datasets, 1723 and 669 strata were created for HCC and MELD exception sub-analysis, respectively. Decompensated-cirrhosis patients (without MELD exception component) accounted for 1863 of the 3459 medium and high MELD recipients (Fig. 2). From this subset, we created 1288 strata.

**Figure 5:**
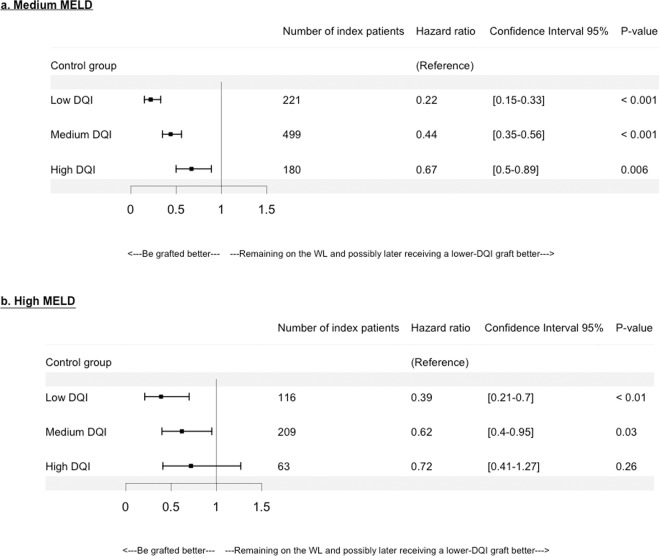
Hazard ratios according to decompensated-cirrhosis and Donor Quality Index (DQI) categories in (**a**) medium MELD: 15 < MELD ≤ 30, and (**b**) high MELD: 30 < MELD, categories. With low DQI: 1.0 < DQI ≤ 1.58, medium DQI: 1.58 < DQI ≤ 2.35, high DQI: 2.35 < DQI. The reference is the control group that consists of patients who remained on the waiting-list (WL) waiting for a potential graft of “lower risk” or “better quality” (i.e. ≤DQI graft) than the one of the index patient.

A description of index patients by sub-groups is presented in Tables 4S, 5S and 6S in Supplementary Information. Among decompensated-cirrhosis patients, those in the medium MELD group experienced a significant SB across each DQI category (Fig. 5a); while for those in the high MELD group, a non-significant SB was observed for patients matched with high DQI grafts (Fig. 5b).

**Figure 6:**
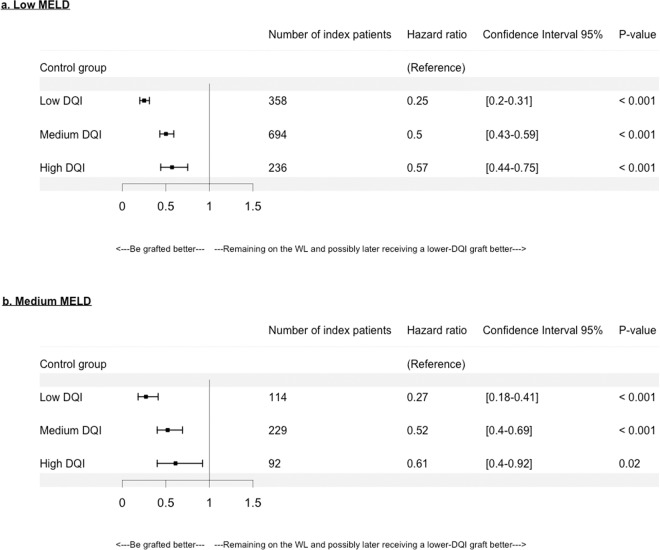
Hazard ratios according to Hepatocellular Carcinoma (HCC) and Donor Quality Index (DQI) categories in (**a**) low MELD: 6 ≤ MELD ≤ 15, and (**b**) medium MELD: 15 < MELD ≤ 30, categories. With low DQI: 1.0 < DQI ≤ 1.58, medium DQI: 1.58 < DQI ≤ 2.35, high DQI: 2.35 < DQI. The reference is the control group that consists of patients who remained on the waiting-list (WL) waiting for a potential graft of “lower risk” or “better quality” (i.e. ≤DQI graft) than the one of the index patient.

For HCC patients, as seen in Fig. 6a,b, a SB was found for all DQI categories, which was similar for low and medium MELD categories.

**Figure 7:**
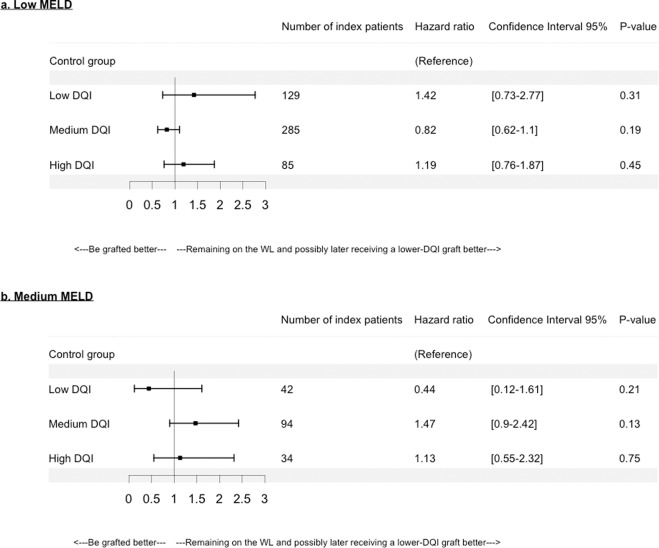
Hazard ratios according to Model for End-stage Liver Disease (MELD) exception and Donor Quality Index (DQI) categories in (**a**) low MELD: 6 ≤ MELD ≤ 15, and (**b**) medium MELD: 15 < MELD ≤ 30, categories. With low DQI: 1.0 < DQI ≤ 1.58, medium DQI: 1.58 < DQI ≤ 2.35, high DQI: 2.35 < DQI. The reference is the control group that consists of patients who remained on the waiting-list (WL) waiting for a potential graft of “lower risk” or “better quality” (i.e. ≤DQI graft) than the one of the index patient.

For MELD exceptions, no significant SB was found, regardless of the DQI category for low MELD (Fig. 7a) and medium MELD (Fig. 7b) categories.

Of note, due to the limited sample size of index patients, the SB within the high MELD category either for HCC or MELD exceptions has not been explored.

## Discussion

Finding the optimal match between a liver graft and a recipient using the sequential stratification method is a realistic way to decrease the effects of organ shortage. The aim of this study was to assess the optimal matching between a recipient and a donor based on the SB according to the quality of the graft and the recipients’ disease severity status. More specifically, for a given donor/recipient couple, we explored whether or not being grafted with a given graft rather than remaining on the WL and possibly later receiving a “better-quality” graft (i.e. ≤DQI graft) was beneficial in terms of survival.

As a whole, a significant SB was observed for all MELD and DQI categories, except for high MELD patients who received “high-risk grafts”. Conversely, “high-risk” grafts appeared to be beneficial for low and medium MELD patients. This was confirmed for decompensated-cirrhosis patients. For MELD exceptions, no significant SB was found. However, HCC patients experienced a SB when receiving “high-risk” grafts.

Our results contrast with those in Schaubel *et al*.^14^, who found that “high-risk” (high-DRI) grafts were beneficial even for high-MELD patients. It should be noted, however, that their results were reported 10 years ago when a different allocation system was in place. Additionally, their patient population has different characteristics from ours; 42% of their patients had hepatitis C virus (HCV) and no efficient antiviral therapies, compared to 23% in our patient population, most of whom are receiving HCV treatment following LT. Conversely, HCC was 2.1% in their sample, while it was 35.2% in ours. Moreover, our study relied on a different metrics. The DRI from Feng *et al*.^7^ was not validated in our population so we opted to use the DQI^11^ instead.

In Schaubel *et al*.^14–16^, patients status after removal from the WL was known. Since we did not have access to information about patient status post WL removal, we considered patients who were removed from the WL due to their worsening condition to have presented the event at their removal date. In clinical practice, these patients are never re-listed and, usually die thereafter. Of note, patients removed from the WL for other reasons (e.g. due to improving of their condition) were censored at their removal date.

We did not match patients on their listing date as proposed by Schaubel *et al*.^14^. Indeed, our population was three fold smaller than in Schaubel *et al*. study^14^ (n = 26,165). However, to mimic an RCT where index and control patients can be wait-listed at the same time, we adjusted on the WL time and on the duration between the listing date and the index patient’s LT date. This variant allowed us to effectively consider all the patients eligible for the graft received by the index patient, which was not the case in Schaubel *et al*.^14^. Moreover, we verified the HR assumption of constancy over time and the consistency of ${\hat{{\boldsymbol{\beta }}}}_{{\boldsymbol{A}}}$.

Due to our sample size, we were not able to form as many MELD categories as in Schaubel *et al*.^14^, in which 12 risk groups were proposed. Indeed, in using so many groups, the control/index patients groups would have been penalized and an obvious lack of power would have been observed.

A complementary analysis was also conducted by MELD and DQI categories in three subsets of important LT indications: decompensated-cirrhosis, HCC, and MELD exceptions. For patients with decompensated-cirrhosis, we found increased LT success among severe patients who received “low-risk” grafts, suggesting that critical patients may benefit from remaining on the WL if they have the potential to receive a “better-quality” graft.

In the growing group of HCC patients, a significant SB was found for low and medium MELD no matter the DQI categories, suggesting that higher risk grafts may be suitable for this group. Our finding sharply contrast with those of other large studies on LT in HCC patients^20,21^ in which a much lower SB was achieved for HCC than for non-HCC patients^22^. However, the design of these observational studies was different from ours^23^. Moreover, in the French allocation system, as in many other allocation systems worldwide, patients with curative therapies (liver resection, ablative radiofrequency) have no access to LT, while LT in intractable or recurrent HCC remaining in the Milano criteria^24^ is favored.

Finally, MELD exceptions did not present any significant SB for either low or medium MELD, which might be due to the heterogeneity of conditions involved in this category. Of note, our population differed from the North American one studied in 2015, where two kinds of exceptions were found: (1) 31.6% of standardized (e.g. HCC), and (2) 8.1% of non-standardized (e.g. cholangitis, refractory ascites, hyponatremia)^25–29^. Many indications in the MELD exception group are guided by the fear that patients would be no longer transplantable (due to metabolic disorders, bacterial resistance, frailty, and hepato-pulmonary syndrome, for example); and by the poor quality of life (QoL) that results from hypoxemia in hepato-pulmonary syndrome, pruritus in biliary diseases, progression of symptoms in genetic diseases, or recurrent encephalopathy. LT generally offers a significant gain in their QoL. An adjustment on the QoL might be studied as a potential improvement of our model; further investigations are required within this subgroup.

In this study, we assumed that DQI and MELD appropriately described donors and recipients, respectively. Moreover, the thresholds taken to construct risk groups are “arbitrary” given the difficulty of establishing risk groups^30^. We also assumed that the covariates used for the matching and those used for adjustment suitably described the outcome. However, we cannot ignore that they may be incomplete. The current allocation system relies on surgeon and center staff expertise to parse through a number of clinical factors in order to match donors to recipients. While our model cannot be prescriptive alone, we suggest that LT teams may be able to incorporate our model to gather further data points that can aid to medical decision making. In this study, we included re-transplanted patients (9.6%), because in the current allocation system, this subpopulation is in competition with other LT candidates. As stated in Schaubel *et al*.^14^, it is important to note that the results we obtained are based on the current allocation system and therefore depend on the matching modalities currently carried out by the ABM.

Some limitations can be noted. First, a lack of power is noted for the high MELD category. Wider intervals were observed compared to other categories since the high MELD group was smaller than the other MELD groups. Second, for decompensated-cirrhosis, HCC or MELD exceptions, the matching was performed according to the studied category, which restricted the number of either index or controls patients. Nevertheless, the numbers of index patients remained satisfactory (Figs. 5, 6 and 7).

## Conclusion

Our aim was to propose an aid to medical decision making for LT donor-recipient matching, including the allocation of “high-risk” grafts, that could be of interest in a context of organ shortage. Given our study population and time period, we showed that “high-risk” grafts provided a significant SB for patients with low or medium MELD and for all HCC patients. “Low-risk” grafts should be preferably allocated to the most severe patients (MELD > 30).

## Methods

We estimated the SB associated with LT in different categories of MELD and DQI using the sequential stratification method derived from Schaubel *et al*.^14–17^. The aim was to determine whether a patient, qualified by his/her disease severity (i.e. MELD), would benefit from receiving an immediate LT with a graft qualified by its DQI (“experimental group”), or should stay on the WL and possibly later receive a “better-quality” or “lower-risk” graft, i.e. a graft with a ≤DQI (“control group”). This method reorganized the observed data in order to be as close as possible to RCT data.

Of note, in the LT literature, the term “survival benefit” describes two distinct concepts. The first, a SB-based liver allocation system, is a combination of urgency- and utility-based systems^31^. The SB is then calculated as the difference between the estimated survival of a post-transplantation model (on recipients’ and donors’ covariates) and the one of a pre-transplantation model (on candidates’ covariates). The SB thus represents a score. This observational approach does not provide any orientation relative to a donor/recipient matching. The other SB approach, the survival improvement, is the gain, in terms of survival, that a given grafted patient (i.e. the index patient) might benefit from, compared to all similar patients remaining on the WL (i.e. control patients)^14–16^. It relies on a stratified model that gathers candidates and recipients. In the present paper, we retained this latter design that mimics an RCT because it is close to an experimental design. It materializes a risk (here a hazard ratio), answering a specific question to a targeted condition; for instance, is a “high-risk” graft suitable for a high-MELD recipient?”

### Sequential stratification: survival benefit in MELD and DQI categories

For each LT, DQI^11^ and MELD^13^ were calculated (see Supplementary Information for more details). They were each categorized into 3 groups. For the DQI, the risk groups are those found in Winter *et al*.^11^ using deciles, where DQI in inversely related to the quality of the graft:

-  Low DQI: 1.0 < DQI ≤1.58,
-  Medium DQI: 1.58 < DQI ≤ 2.35,
-  High DQI: 2.35 < DQI (i.e. considered as “high-risk” or “low-quality” grafts).

Of note, the DQI score includes the five following donor covariates: age, cause of death (trauma, cerebrovascular accident, anoxia and other), length of Intensive Care Unit (ICU) stay, lowest modification of diet in renal disease creatinine clearance value, and liver type (total/split).

The retained MELD categories according to the ABM (https://www.agence-biomedecine.fr/annexes/bilan2016/donnees/organes/05-foie/synthese.htm#) were:

- Low MELD: 6 ≤ MELD ≤ 15,
- Medium MELD: 15 < MELD ≤ 30,
- High MELD: MELD > 30.

A stratum was created for each LT observed in the dataset (Fig. 3). Each stratum included the transplanted patient (who is the index patient) and all the matched “control” patients, who:

- were active on the WL (not grafted, still alive, not removed from the WL, not under temporary contraindication, not lost to follow-up);
- were in the same MELD group as the index patient at the time of LT (the MELD is calculated at the time of the LT of reference for the index patient and the control patients);
- were aged within ±5 years of the index patient;
- had the same high emergency status (i.e. acute liver failure, primary non-function) as the index patient;
- and were ABO compatible with the graft received by the index patient.

Once included in a stratum, “control” patients were censored only if they had received a transplant with a DQI higher than the index transplant. Thus, the control group consists of patients who remained on the WL waiting for a potential “better-quality” graft (i.e. ≤DQI graft) than the one received by the index patient. They were then censored at their own transplant date.

Analogous with RCT data, all patients from a same stratum share “the same ${t}_{0}$”. In a stratum, follow-up began when the index patient was transplanted and ended when the first instance of any of the following events occurred: loss to follow-up, removal from the WL, death on WL, graft loss (re-transplantation), death after LT, LT with a DQI higher than the index transplant, or end of the study (December 31, 2016). The outcome was defined as a composite outcome, and composed of death on the WL, removal from WL due to worsening of patient’s condition, death after LT, or graft loss (re-transplantation).

Strata were then combined, and a stratified Cox regression model was fitted. The hazard function was:

$${\lambda }_{i(\ell )}(t,\,{\boldsymbol{\beta }})={\lambda }_{0(\ell )}(t){e}^{{{\boldsymbol{\beta }}}_{{\boldsymbol{A}}}^{{\boldsymbol{T}}}({\boldsymbol{M}}{\boldsymbol{E}}{\boldsymbol{L}}{\boldsymbol{D}}\times {\boldsymbol{D}}{\boldsymbol{Q}}{\boldsymbol{I}}{)}_{i(\ell )}+{{\boldsymbol{\beta }}}_{{\boldsymbol{B}}}^{{\boldsymbol{T}}}{{\boldsymbol{Z}}}_{{\boldsymbol{i}}{\boldsymbol{(}}{\boldsymbol{\ell }}{\boldsymbol{)}}}},$$

where ${\boldsymbol{\beta }}=({{\boldsymbol{\beta }}}_{{\boldsymbol{A}}}^{{\boldsymbol{T}}},\,{{\boldsymbol{\beta }}}_{{\boldsymbol{B}}}^{{\boldsymbol{T}}})$ the coefficients, $\,{{\boldsymbol{Z}}}_{{\boldsymbol{i}}}(t)$ the covariates vector, $\ell$ the stratum, *i* the patient, and ${{\boldsymbol{\beta }}}_{{\boldsymbol{A}}}={({\beta }_{1},\ldots ,{\beta }_{9})}^{T}$ represent the coefficients of interest to estimate, corresponding to the $({\boldsymbol{M}}{\boldsymbol{E}}{\boldsymbol{L}}{\boldsymbol{D}}\times {\boldsymbol{D}}{\boldsymbol{Q}}{\boldsymbol{I}}{)}_{i(\ell )}$ covariate, which takes the value 1 for the corresponding combinations between index patient MELD and donor DQI, and takes the value 0 otherwise (control patients matched: patients remained on the WL waiting for a potential “better-quality” graft).

The covariates vector ${{\boldsymbol{Z}}}_{i(\ell )}$ included the LT candidate’s sex, body mass index, diabetes status, whether the candidate had a previous transplantation, status at listing (ICU, hospitalized but not in ICU, or at home), whether the candidate was on dialysis, decompensated-cirrhosis, HCC and MELD exception. Moreover, for each patient included in a stratum (as presented above), we computed the total time spent on the WL and the time spent on the WL before the date of the index patient’s LT (by definition, these two durations are identical for the index patient). Finally, we created a covariate “region-matching”, which we coded 0 if the index patient and the matched patients belonged to the same region; and 1 otherwise.

The SB was estimated through HRs in each MELD and DQI category

We assumed that the censorship was conditionally independent of the outcome knowing $\,{{\boldsymbol{Z}}}_{{\boldsymbol{i}}}(t)$ and the strata ${s}_{i}$.

The HRs allowed the comparison of patients, for each MELD and DQI category, with the group of candidates remaining on the WL and waiting for a potential graft of “better quality”, i.e. with a ≤DQI.

Of note, we also verified the assumptions of the model, namely the normality of the ${\beta }_{1},\,\ldots ,\,{\beta }_{9}$ coefficients and the constancy of the HRs over time^15^, see Supplementary Methods in Supplementary Information for more details.

### Survival benefit in decompensated-cirrhosis, HCC and MELD exception patients

MELD does not have the same meaning for each indication of LT. Therefore, we studied MELD-DQI matching in the 3 main indications: decompensated-cirrhosis, HCC and MELD exception.

In patients with decompensated-cirrhosis, the allocation score is mostly based on the MELD score. These patients were, by the definition given above, distributed only among the medium and high MELD groups.

In HCC patients, mortality risk is not reflected by MELD score in clinical practice. In 2013, 75.7% of HCC patients had a MELD score lower than 15, and 90.2% had a MELD score lower than 20 (https://www.agence-biomedecine.fr/annexes/bilan2013/donnees/organes/05-foie/synthese.htm). These subgroups represented 30 to 35% of the WL in the last five years (see Supplementary Information for more details).

For patients with a MELD exception, the MELD score is not considered representative of their clinical severity. Thus, they receive extra-points, either at listing or progressively (i.e. at 3, 6, 9 or 12 months), placing them directly in competition with patients registered either for HCC and waiting for more than 15 months, or for decompensated-cirrhosis (https://www.agence-biomedecine.fr/annexes/bilan2013/donnees/organes/05-foie/synthese.htm).

Therefore, we explored the SB in patients with decompensated-cirrhosis, HCC or MELD exception, using the same method plus more an indication matching (i.e. taking into consideration the date at which the MELD exception occurred). Of note, when dealing with decompensated-cirrhosis or HCC, patients (i.e. index or controls) cannot be MELD exception at the index patient’s LT. Controls were matched on the index patient’s diagnosis.

All analyses and Figs. 2 and 4 to 7 were produced using R software, version 3.3.0^32^ with the *survival* package (https://www.R-project.org/).

### Independent ethics committee

The data was provided by the “Agence de la Biomédecine” (ABM) in the context of the OPTIMATCH program. This program has been authorized and funded by the Ministry of Health. In France, the ABM is in charge of managing the waiting list and distributing the grafts (http://www.agence-biomedecine.fr/). It hosts the data relating to donors and recipients. The study was conducted according to the approval given by the “Comité de Protection des Personnes” of Paris Ile-de-France which is an independent Ethics Committee. It was also approved by the “Comité Consultatif pour le Traitement de l’Information en matière de Recherche dans le domaine de la Santé”. Authorization was also obtained from the “Commission Nationale de l’Informatique et des Libertés” (agreement No. 915206), authorized to approve study on humans (https://www.cnil.fr/en/home). We obtained the de-identified data from the ABM; patients were not directly involved.

## Supplementary information

Supplementary information.

## Data Availability

The data that support the findings of this study are available upon request from the “Agence de la Biomédecine” (https://www.agence-biomedecine.fr/demande-acces-donnees-cristal).
