# Supplementary material for: Matching Graft Quality to Recipient’s Disease Severity Based on the Survival Benefit in Liver Transplantation
Source: Sci Rep. 2020 Mar 5;10:4111. doi: 10.1038/s41598-020-60973-9 (PMC7057972; doi:10.1038/s41598-020-60973-9)
Supplement: Supplementary file 1 — Supplementary information. [file 41598_2020_60973_MOESM1_ESM.pdf]

Supplementary Information: Matching Graft Quality to Recipient's Disease Severity Based on the Survival Benefit in Liver Transplantation

Audrey Winter, PhD, Cyrille Féray, MD PhD, Corinne Antoine, MD, Daniel Azoulay, MD PhD, Jean-Pierre Daurès, MD PhD, Paul Landais, MD PhD

Liver transplantation, allocation process in France

In the early years of liver transplantation (LT), allocation was a clinician-guided decision. The major factor driving allocation was time elapsed on the waiting list, which resulted in premature and even futile transplantation and high mortality among the sickest candidates. In this context, a prognostic score called Model for End-Stage Liver Disease (MELD)[1] was published, which became the metric of choice for reflecting the severity of liver disease, and was found to be able to accurately predict 3-month mortality.

The MELD score (including serum creatinine, bilirubin and coagulation time; MELD = 3.78*ln(serum bilirubin[mg/dl]) + 11.2*ln(International Normalized Ratio) + 9.57* ln(serum creatinine[mg/dl]) + 6.43) was adopted in 2002 by the American Organization for Organ Sharing (UNOS) for allocation of liver grafts to cirrhotic candidates. Using MELD, it became possible to rank wait list candidates according to risk of death, irrespective of wait time (medical emergency)[2]. In the United States the adoption of the MELD score has been followed by a reduction of mortality on the waiting list[3], and a dramatic decrease in futile indications for LT.

In France in 2007, "l'Agence de la Biomédecine" (ABM) established a new nation-wide allocation system called "Score National Foie". The system is based on a policy of 'sickest first', whereby patients who are at higher risk of death are given higher wait list priority. The system separates patients according to two categories: super urgent indications, such as acute liver failure, which

receive the highest national priority; and elective indications, which are managed once super urgent indications have been transplanted.

Among elective indications, the system further separates patients who are listed for decompensated cirrhosis, whose 3-month risk of death is accurately predicted by the MELD score[4], and those listed for primary liver tumors. In both cases, each listed patient receives a score (score national foie) ranging from 0 to 1000 points which defines their priority on the wait list (higher scores result in shorter wait time). In patients with decompensated cirrhosis, those with the highest MELD score are served first and allocation only depends on MELD, directly converted to the 0/1000 points scale. For this subset, score is not influenced by time elapsed on the wait list. By contrast, in patients listed for HCC with usually preserved liver function (median MELD score 12), the risk of death on wait list is limited and patients acquire points over a 12-month period to get the highest priority after one year on the wait list, competing at that time with cirrhotic patients with high MELD score.

The Donor Quality Index (DQI)[5]

Since we showed that the existing Donor Risk Index (DRI[6] and ET-DRI[7]) did not fit the French database[8], the DQI was developed, according to the TRIPOD[9], using 3961 liver transplantations performed in France between January 4, 2009 and December 31, 2013. It was based on a Cox model with adjustment on recipient characteristics. The retained donor covariates were age, cause of death (COD), length of intensive care unit (ICU) stay, lowest MDRD creatinine clearance, and liver type.

Several recipient covariates were retained for adjustment: re-transplantation, being on dialysis, status at time of LT, presence of hepatitis C virus antibody, diabetes, decompensated cirrhosis, MELD exceptions, and donor and recipient's blood groups.

Hence the proposed DQI score was as follows:

DQI = exp (0.28 (1 if donor age > 69 years; 0 otherwise)

+ 0.06 (1 if COD is “other”; 0 otherwise)

+ 0.30 (1 if COD is “cerebrovascular accident (CVA)”; 0 otherwise)

+ 0.11 (1 if COD is “trauma”; 0 otherwise)

+ 0.24 (1 if ICU stay is ≤ 4 days; 0 otherwise)

+ 0.22 (1 if the lowest MDRD creatinine clearance < 60 ml/min/1.73m^2; 0 otherwise)

+ 0.05 (1 if the lowest MDRD creatinine clearance ≥ 60 ml/min/1.73m^2 and < 90 ml/min/1.73m^2; 0 otherwise)

+ 0.39 (1 if split or partial liver; 0 otherwise)).

Three discriminative risk groups were obtained according to the following values: 1.00 < DQI ≤ 1.58; 1.58 < DQI ≤ 2.35 and DQI > 2.35, comprising 34.1%, 56.8% and 9.1% patients, respectively **(Figure 1S)**.

An external validation[8–10] was performed in the validation dataset, which contained 1048 LTs performed in France between January 1, 2014 and December 31, 2014. Both the apparent calibration and the discrimination were preserved.

Supplementary Methods

Consistency/ normality assumption

The consistency of $\beta_1, \ldots, \beta_9$ was checked through the method described in Schaubel et al.[11]. A bootstrap method from Efron [12] was used to estimate the distribution of $\hat{\beta}$ empirically. Two hundred resamples were made. Due to the dataset structure (with strata) obtained after application of the sequential stratification method, the bootstrap was performed as follows:

1- A sampling with replacement was performed among index patients of the nine MELD and DQI categories. The same number of index patients as in the original database was sampled.

The proportions were not respected in the different MELD and DQI categories during the sampling. Nevertheless, the sampling repetition generated a sample representative of the general population.

2- Another sampling was then performed for the control group. The number of patients depended on the stratum. For each stratum, we maintained the same number of control patients it originally contained.

3- The two databases were then merged in order to create the bootstrap database.

4- A stratified Cox model, as previously presented, was applied to the bootstrap database and the $\widehat{\boldsymbol{\beta}}_{A,b}$ were estimated.

Steps 1 to 4 were repeated 200 times. Diagnostic plots (histograms, q/q plots) of the $\{\hat{\beta}_{i,b}$; b = 1, …, 200 and i = 1, …, 9} were used to check the normality assumption.

Constancy of the HRs over time assumption

As in Schaubel et al. [11], in order to verify this assumption, we estimated the HRs for transplants occurring in years 1 and 2+ of follow-up: $(\beta_1^1, \ldots, \beta_9^1)$ for year 1 and $(\beta_1^{2+}, \ldots, \beta_9^{2+})$ for year 2+.

Supplementary Results

Consistency/ normality assumption

We then tested the consistency of $\widehat{\boldsymbol{\beta}}_{A}$ using 200 simulations. The results of the 200 simulations obtained are given in **Table 1S**, as well as the P-values and confidence intervals obtained by normal approximation.

The results obtained are similar to those obtained in **Figure 3**. We then graphically verified the normality assumption which was consistent.

Constancy over time

We verified whether the HRs were constant across time. We then fitted a model by splitting each MELD and DQI categories into two parts; firstly, LT occurring in the first year on the WL; secondly, LT occurring in the second year or beyond. The results obtained are presented in **Table 2S**. For the first year we obtained results consistent with the one presented above, namely a significant survival benefit for each category of MELD and DQI except for the “high MELD and high DQI category” (i.e. non-significant survival benefit).

For the second year, the survival benefit was not constant for all categories along with the waiting period. Indeed, three scenarios occurred:

- Persistent survival benefit for “Low MELD and High DQI”, “Medium MELD and Low DQI”, and “Medium MELD and Medium DQI” categories.
- Increasing survival benefit for “Low MELD and Low DQI”, and “Low MELD and Medium DQI” categories.
- Non-significant HRs for four categories (i.e. all “high MELD” categories and “Medium MELD and High DQI” category).

This assumption was thus not valid for all MELD and DQI categories. HRs seemed dependent on the LT time. We were able to test this hypothesis only for LTs at more or less one year after listing. A lack of power did not enable an appropriate interpretation of these results. Indeed, splitting into two groups in order to consider LTs performed before or after 1-year post wait-listing led to few index patients for some MELD and DQI categories. Furthermore, a selection bias might be present since patients grafted after more than one year on the WL might be “less ill” than patients grafted in their first year on the WL.

References

1. Malinchoc, M. *et al.* A model to predict poor survival in patients undergoing transjugular intrahepatic portosystemic shunts. *Hepatology* **31**, 864–871 (2000).
2. Kamath, P. S. *et al.* A model to predict survival in patients with end-stage liver disease. *Hepatology* **33**, 464–470 (2001).
3. Weismüller, T. J. *et al.* The introduction of MELD-based organ allocation impacts 3-month survival after liver transplantation by influencing pretransplant patient characteristics. *Transplant International* **22**, 970–978 (2009).
4. Wiesner, R. *et al.* Model for end-stage liver disease (MELD) and allocation of donor livers. *Gastroenterology* **124**, 91–96 (2003).
5. Winter, A. *et al.* A Donor Quality Index for liver transplantation: development, internal and external validation. *Scientific Reports* **8**, 9871–9884 (2018).
6. Feng, S. *et al.* Characteristics associated with liver graft failure: the concept of a donor risk index. *American Journal of Transplantation* **6**, 783–790 (2006).
7. Braat, A. *et al.* The Eurotransplant Donor Risk Index in Liver Transplantation: ET-DRI. *American Journal of Transplantation* **12**, 2789–2796 (2012).
8. Winter, A. *et al.* External validation of the Donor Risk Index and the Eurotransplant Donor Risk Index on the French liver transplantation registry. *Liver International* **37**, 1229–1238 (2017).
9. Moons, K. G. *et al.* Transparent Reporting of a multivariable prediction model for Individual Prognosis Or Diagnosis (TRIPOD): explanation and elaboration. *Annals of internal medicine* **162**, W1–W73 (2015).
10. Royston, P. & Altman, D. G. External validation of a Cox prognostic model: principles and methods. *BMC medical research methodology* **13**, 33 (2013).
11. Schaubel, D. E., Wolfe, R. A. & Port, F. K. A Sequential Stratification Method for Estimating the Effect of a Time-Dependent Experimental Treatment in Observational Studies. *Biometrics* **62**, 910–917 (2006).
12. Efron, B. *The jackknife, the bootstrap and other resampling plans*. vol. 38 (SIAM, 1982).

Supplementary Table Legends

- **Table 1S** – Proportion of non-transplanted grafts in the 2009-2014 period and related causes.
- **Table 2S** – Hazard ratios by Model for End-stage Liver Disease (MELD) categories for all Donor Quality Index (DQI) after 200 bootstrap loops. The reference is the control group that consists of patients who remained on the waiting-list (WL) waiting for a potential graft of "better quality" (i.e. a lower-DQI graft) than the one of the index patient. Hazard ratios are given with their 95% confidence intervals. ***$p<0.001$; **$p<0.01$; *$p<0.05$; p: NS otherwise.
- **Table 3S** – Hazard ratios estimated by categories of Model for End-stage Liver Disease (MELD) and Donor Quality Index (DQI) for liver transplants occurring during year 1 and 2+ after the listing. The reference is the control group that consists of patients who remained on the waiting-list (WL) waiting for a potential graft of "better quality" (i.e. a lower-DQI graft) than the one of the index patient. Hazard ratios are given with their 95% confidence intervals. ***$p<0.001$; **$p<0.01$; *$p<0.05$; p: NS otherwise.
- **Table 4S** – Index patient characteristics in decompensated-cirrhosis sub-group by medium, $15 < \text{MELD} \leq 30$, and high, $\text{MELD} > 30$, Model for End-stage Liver Disease (MELD) categories. "Mean (standard deviation), median" is reported for quantitative covariates; "number (percentage)" is reported for qualitative covariates.
- **Table 5S** – Index patient characteristics in hepatocellular-carcinoma sub-group by low, $6 \leq \text{MELD} \leq 15$, and medium, $15 < \text{MELD} \leq 30$, Model for End-stage Liver Disease (MELD) categories. "Mean (standard deviation), median" is reported for quantitative covariates; "number (percentage)" is reported for qualitative covariates.
- **Table 6S** – Index patient characteristics in Model for End-stage Liver Disease (MELD) exception sub-group by low, $6 \leq \text{MELD} \leq 15$, and medium, $15 < \text{MELD} \leq 30$, Model for End-stage Liver Disease (MELD) categories. "Mean (standard deviation), median" is reported for quantitative covariates; "number (percentage)" is reported for qualitative covariates.

Supplementary Figure Legends

- **Figure 1S** – Survival curve using Kaplan Meier estimate for the three risk groups of the Donor Quality Index score.

Table 1S – Proportion of non-transplanted grafts in the 2009-2014 period and related causes.

**Causes:** **Year:**	**2009**	**2010**	**2011**	**2012**	**2013**	**2014**
Macroscopic steatosis (with or without biopsy)	38	41	40	35	28	35
Poor graft quality	19	23	32	25	17	20
Tumor (or suspicion)	2	5	9	10	9	12
Recipient cause	1	1	0	3	1	2
Other Causes*	9	7	4	5	11	4
**Total**	**69**	**77**	**85**	**78**	**66**	**73**
**Proportion among collected grafts (%)**	**6.3**	**6.7**	**7**	**6.3**	**5.1**	**5.4**

* logistic impediments, damaged graft, technical difficulties of surgery, anatomical difficulties, cardiac arrest during organ removal, etc)

Table 2S – Hazard ratios by Model for End-stage Liver Disease (MELD) categories for all Donor Quality Index (DQI) after 200 bootstrap loops.

The reference is the control group that consists of patients who remained on the waiting-list (WL) waiting for a potential graft of "better quality" (i.e. a lower-DQI graft) than the one of the index patient. Hazard ratios are given with their 95% confidence intervals. ****p*<0.001; ***p*<0.01; **p*<0.05; *p*: NS otherwise.

DQI<br>MELD	Low<br>1.0-1.58	Medium<br>1.59-2.35	High<br>>2.35
**Low**<br>**6-15**	0.26***<br>[0.21-0.31]	0.52***<br>[0.46-0.58]	0.64***<br>[0.53-0.78]
**Medium**<br>**16-30**	0.22***<br>[0.18-0.28]	0.49***<br>[0.44-0.56]	0.70***<br>[0.58-0.86]
**High**<br>**> 30**	0.40*<br>[0.16-0.99]	0.53**<br>[0.33-0.86]	0.59<br>[0.31-1.14]

Table 3S – Hazard ratios estimated by categories of Model for End-stage Liver Disease (MELD) and Donor Quality Index (DQI) for liver transplants occurring during year 1 and 2+ after the listing.
The reference is the control group that consists of patients who remained on the waiting-list (WL) waiting for a potential graft of "better quality" (i.e. a lower-DQI graft) than the one of the index patient. Hazard ratios are given with their 95% confidence intervals. ***p<0.001; **p<0.01; *p<0.05; p: NS otherwise.

	MELD \ DQI	Low 1.0-1.58	Medium 1.59-2.35	High >2.35
First year	**Low 6-15**	0.31*** [0.25-0.39]	0.57*** [0.49-0.66]	0.64*** [0.50-0.82]
	**Medium 16-30**	0.26*** [0.20-0.33]	0.51*** [0.43-0.60]	0.68*** [0.55-0.85]
	**High > 30**	0.44*** [0.29-0.68]	0.69** [0.55-0.89]	0.73 [0.51-1.05]
Second year and more	**Low 6-15**	0.19*** [0.13-0.27]	0.43*** [0.34-0.55]	0.66* [0.48-0.93]
	**Medium 16-30**	0.27*** [0.15-0.49]	0.60** [0.41-0.88]	1.01 [0.56-1.81]
	**High > 30**	2.32 [0.43-12.59]	0.16 [0.02-1.19]	1.62 [0.50-5.27]

Table 4S – Index patient characteristics in decompensated-cirrhosis sub-group by medium, 15 < MELD ≤ 30, and high, MELD > 30, Model for End-stage Liver Disease (MELD) categories. "Mean (standard deviation), median" is reported for quantitative covariates; "number (percentage)" is reported for qualitative covariates.

	**Medium MELD (N=900)**	**High MELD (N=388)**
Age	53.1 (8.9), 53.9	54.3 (7.6), 55.1
Female	254 (28.2%)	108 (27.8%)
Body mass index	25.7 (4.7), 25	27.2 (5.9), 26.3
Diabetes	159 (17.7%)	58 (14.9%)
Previous transplantation	17 (1.9%)	14 (3.6%)
On dialysis	5 (0.6%)	48 (12.4%)
Status at listing:		
Intensive Care Unit	62 (6.9%)	165 (42.5%)
Hospital	158 (17.6%)	124 (32%)
Home	680 (75.6%)	99 (25.5%)
MELD at listing	22.6 (4.1), 22	31.7 (7), 33
MELD at transplantation	24.1 (3.9), 24	36.9 (3.3), 38
HBV positives at transplantation	42 (4.7%) *	24 (6.3%) †
HCV positives at transplantation	168 (18.9%) **	57 (15.2%) ††
Waiting time (in days)	102.7 (141.7), 57	48.9 (117.5), 11.5
Cold ischemia time (in minutes)	479 (207.8), 458	483.1 (204.5), 459.5
Donor Quality Index	1.9 (0.5), 1.8	1.8 (0.5), 1.8

* 10 missing data
** 12 missing data
† 10 missing data
†† 14 missing data

Table 5S – Index patient characteristics in hepatocellular-carcinoma sub-group by low, $6 \leq$ MELD $\leq$ 15, and medium, $15 <$ MELD ≤ 30, Model for End-stage Liver Disease (MELD) categories. "Mean (standard deviation), median" is reported for quantitative covariates; "number (percentage)" is reported for qualitative covariates.

	**Low MELD (N=1288)**	**Medium MELD (N=435)**
Age	57.7 (7), 58.7	58.2 (6.2), 59.5
Female	139 (10.8%)	65 (14.9%)
Body mass index	26.9 (4.3), 26.6	27.6 (4.8), 27.1
Diabetes	420 (32.6%)	138 (31.7%)
Previous transplantation	10 (0.8%)	4 (0.9%)
On dialysis	-	1 (0.2%)
Status at listing:		
Intensive Care Unit	5 (0.4%)	8 (1.8%)
Hospital	31 (2.4%)	37 (8.5%)
Home	1252 (97.2%)	390 (89.7%)
MELD at listing	10.1 (3.1), 9	17 (4.9), 17
MELD at transplantation	9.8 (2.7), 9	20.7 (4.1), 20
Waiting time (in days)	323.8 (217.5), 311	205.6 (164.9), 164
Cold ischemia time (in minutes)	504.5 (284), 480	500.3 (255.9), 480
Donor Quality Index	1.9 (0.5), 1.8	1.9 (0.5), 1.8
Number of tumors	2 (1.2), 2 *	1.9 (1.2), 2 †
Largest tumor (in cm)	3.2 (2.8), 2.6 **	2.8 (2), 2.4 †
α-foetoprotein score > 2	87 (10.2%) ***	20 (6.4%) ††

* 24 missing data
** 33 missing data
*** 436 missing data
† 20 missing data
†† 123 missing data

Table 6S – Index patient characteristics in Model for End-stage Liver Disease (MELD) exception sub-groups by low, $6 \leq$ MELD ≤ 15, and medium, $15 <$ MELD ≤ 30, Model for End-stage Liver Disease (MELD) categories. "Mean (standard deviation), median" is reported for quantitative covariates; "number (percentage)" is reported for qualitative covariates.

	**Low MELD (N=499)**	**Medium MELD (N=170)**
Age	52.3 (10.6), 54.1	54.8 (8.3), 56.0
Female	180 (36.1%)	50 (29.4%)
Body mass index	24.3 (4.3), 23.8	24.8 (4.9), 24.3
Diabetes	97 (19.4%)	59 (34.7%)
On dialysis	2 (0.4%)	1 (0.6%)
Status at listing:		
Intensive Care Unit	17 (3.4%)	6 (3.5%)
Hospital	59 (11.8%)	31 (18.2%)
Home	423 (84.8%)	133 (78.2%)
Hepatocellular carcinoma (HCC)	27 (5.4%)	11 (6.5%)
Decompensated cirrhosis	51 (10.2%)	71 (41.8%)
Re-transplantation	67 (13.4%)	37 (21.8%)
Non-HCC liver tumor	50 (10.0%)	1 (0.6%)
Cirrhosis	308 (61.7%)	118 (69.4%)
Non-cirrhotic liver disease	66 (13.2%)	13 (7.6%)
MELD at listing	11.3 (4.0), 11	16.8 (4.0), 17
MELD at transplantation	10.2 (3.1), 10	19.8 (3.5), 19
Waiting time (in days)	236.4 (221.1), 197	249.3 (215.5), 208
Cold ischemia time (in minutes)	507.5 (367.8), 460	478.6 (154.8), 480
Donor Quality Index	1.9 (0.5), 1.8	1.9 (0.5), 1.8

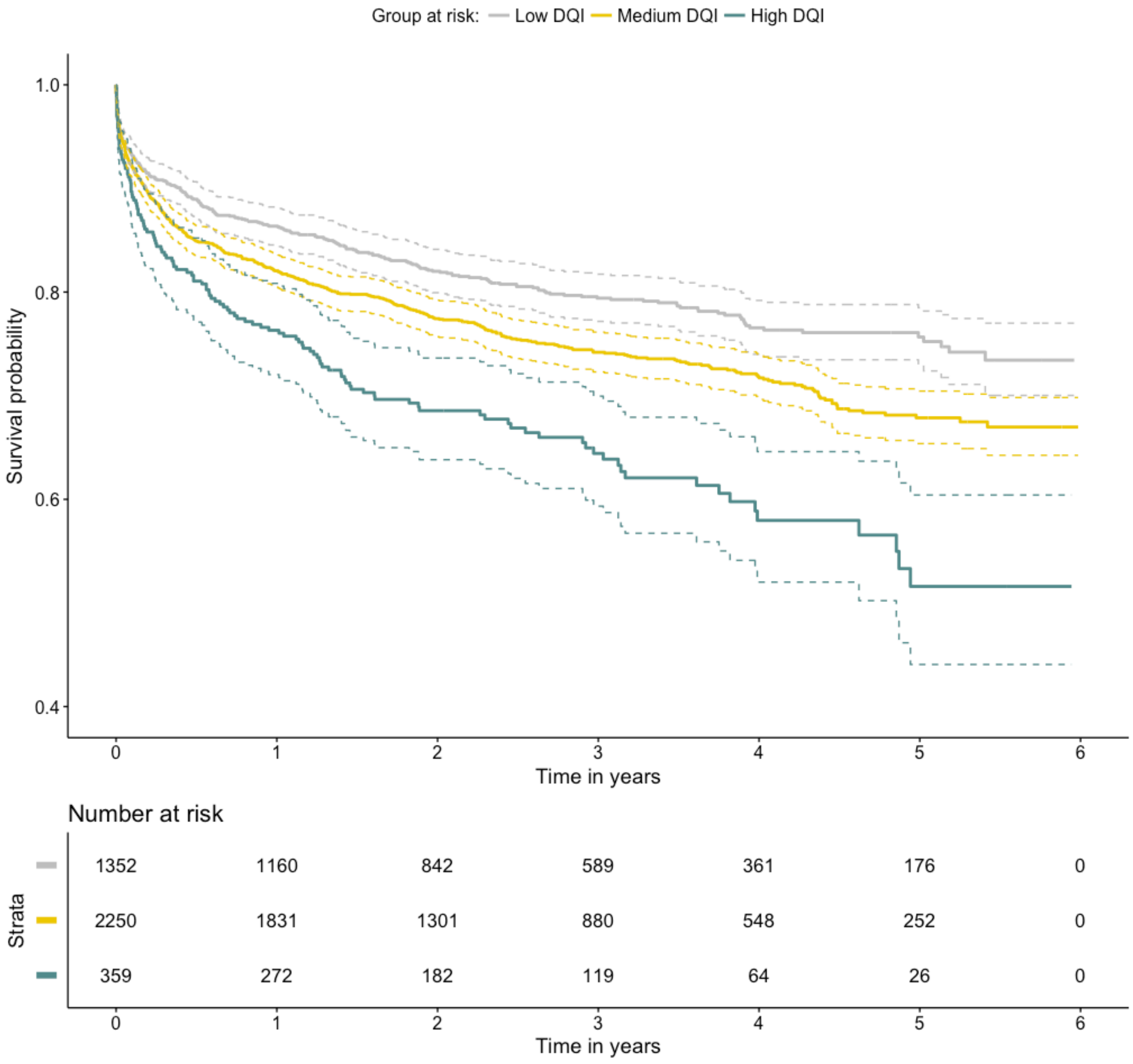

Figure 1S – Survival curve using Kaplan Meier estimate for the three risk groups of the Donor Quality Index score.
